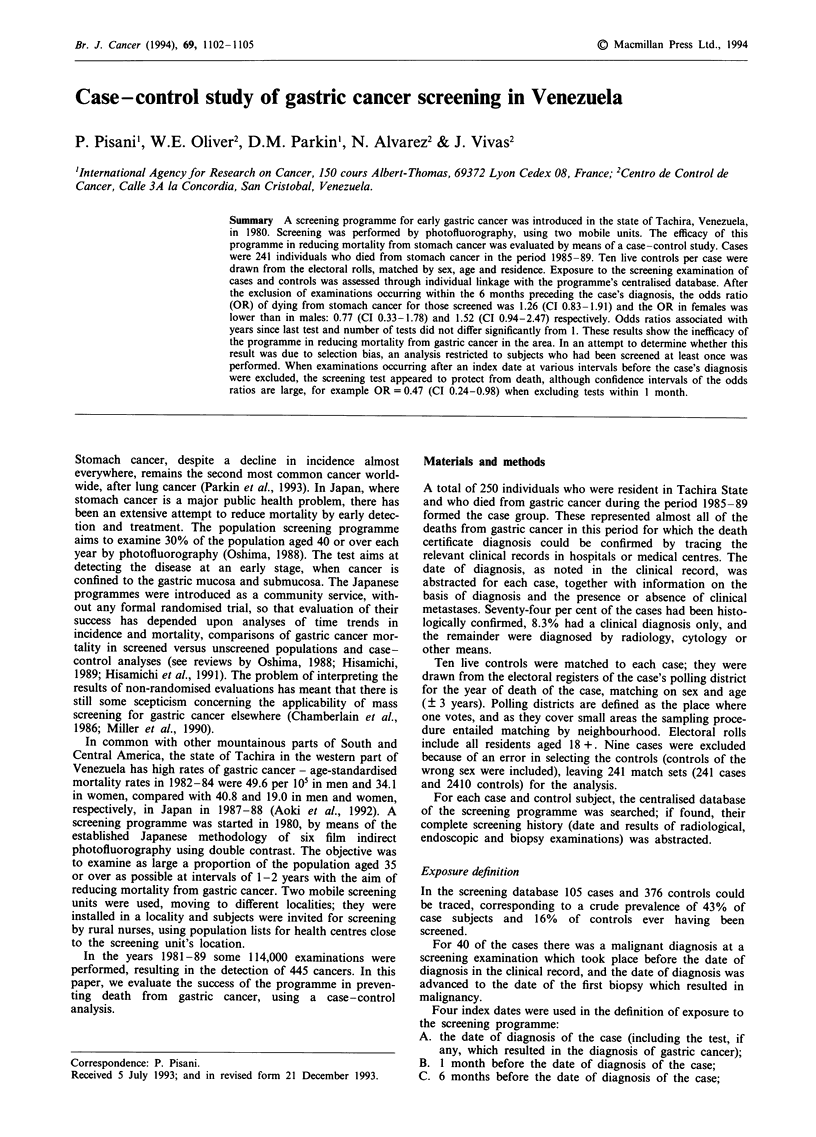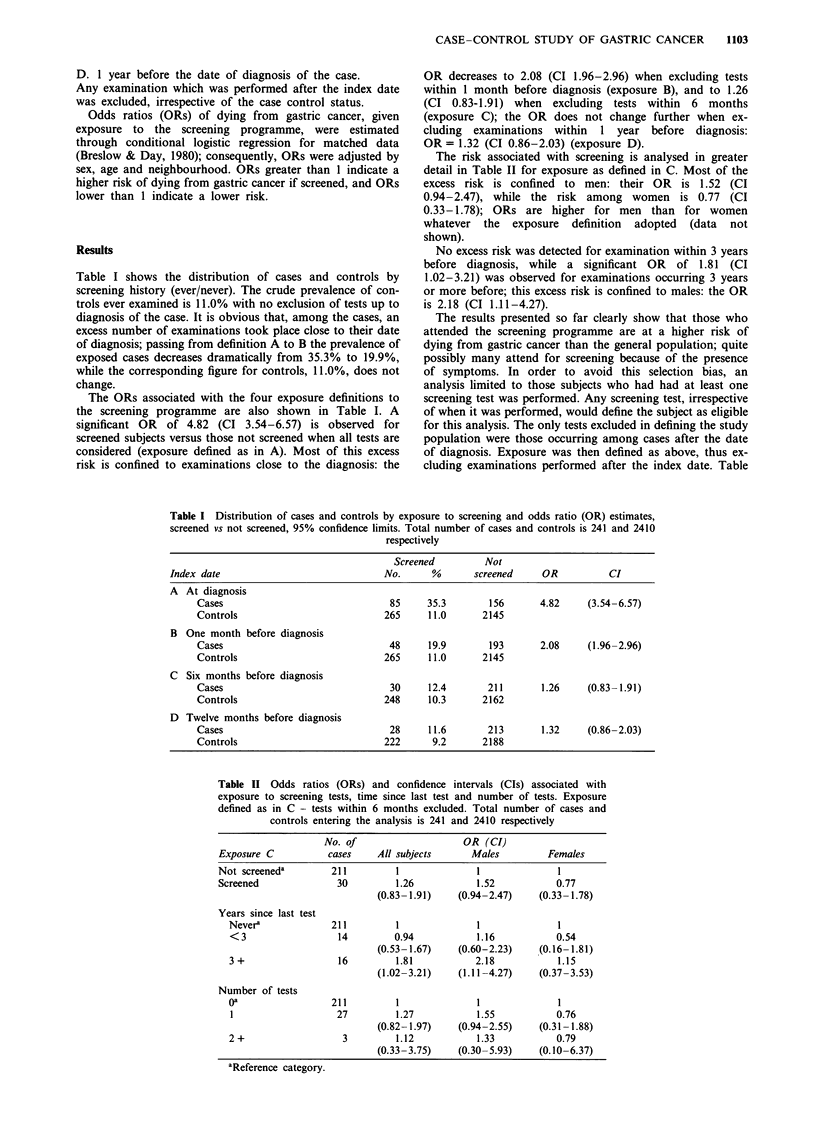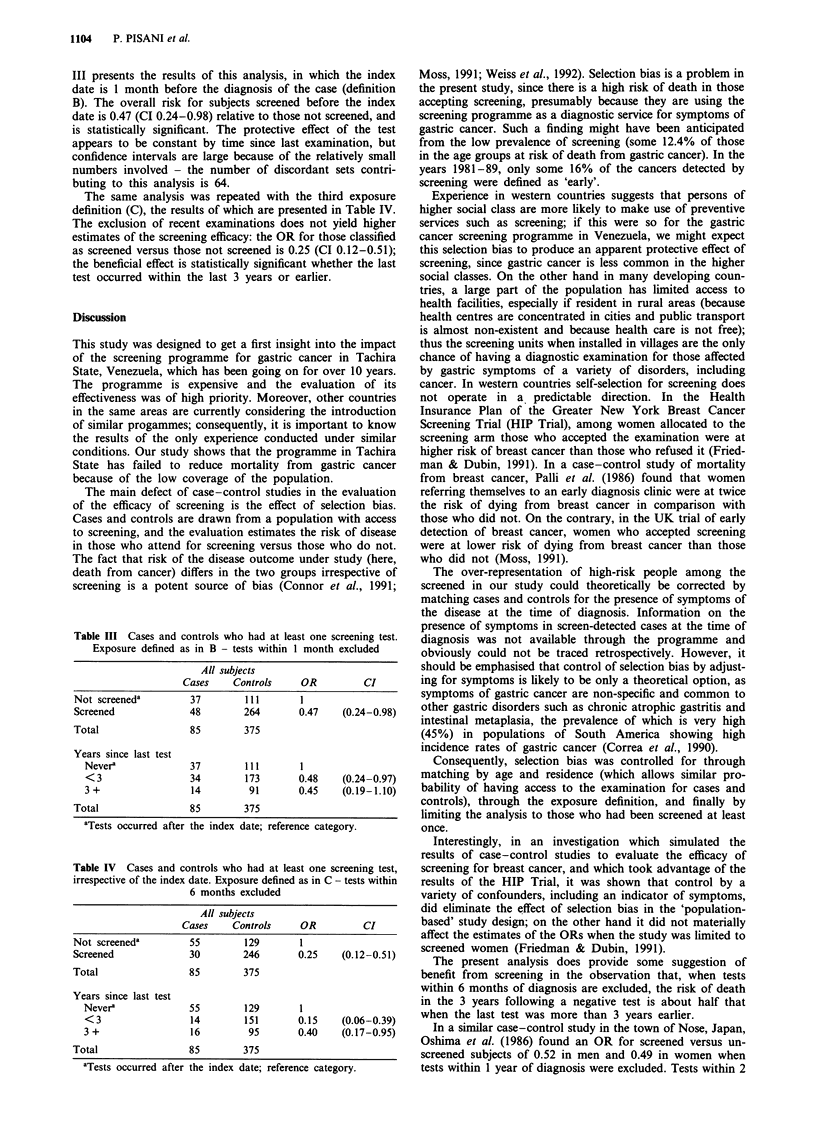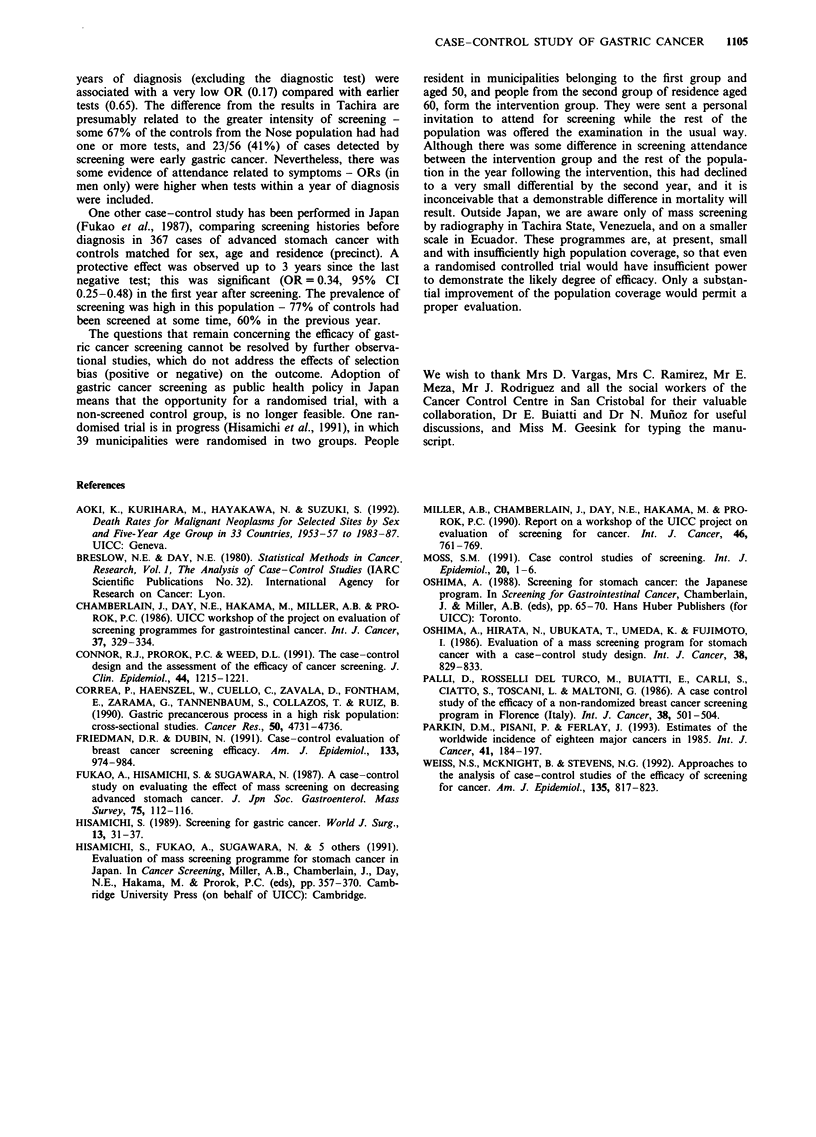# Case-control study of gastric cancer screening in Venezuela.

**DOI:** 10.1038/bjc.1994.216

**Published:** 1994-06

**Authors:** P. Pisani, W. E. Oliver, D. M. Parkin, N. Alvarez, J. Vivas

**Affiliations:** International Agency for Research on Cancer, Lyon, France.

## Abstract

A screening programme for early gastric cancer was introduced in the state of Tachira, Venezuela, in 1980. Screening was performed by photofluorography, using two mobile units. The efficacy of this programme in reducing mortality from stomach cancer was evaluated by means of a case-control study. Cases were 241 individuals who died from stomach cancer in the period 1985-89. Ten live controls per case were drawn from the electoral rolls, matched by sex, age and residence. Exposure to the screening examination of cases and controls was assessed through individual linkage with the programme's centralised database. After the exclusion of examinations occurring within the 6 months preceding the case's diagnosis, the odds ratio (OR) of dying from stomach cancer for those screened was 1.26 (CI 0.83-1.91) and the OR in females was lower than in males: 0.77 (CI 0.33-1.78) and 1.52 (CI 0.94-2.47) respectively. Odds ratios associated with years since last test and number of tests did not differ significantly from 1. These results show the inefficacy of the programme in reducing mortality from gastric cancer in the area. In an attempt to determine whether this result was due to selection bias, an analysis restricted to subjects who had been screened at least once was performed. When examinations occurring after an index date at various intervals before the case's diagnosis were excluded, the screening test appeared to protect from death, although confidence intervals of the odds ratios are large, for example OR = 0.47 (CI 0.24-0.98) when excluding tests within 1 month.


Br. J. Cancer (1994), 69, 1102–1105 © Macmillan Press Ltd., 1994

### Case–control study of gastric cancer screening in Venezuela

P. Pisani[1], W.E. Oliver[2], D.M. Parkin[1], N. Alvarez[2] & J. Vivas[2]

[1]*International Agency for Research on Cancer, 150 cours Albert-Thomas, 69372 Lyon Cedex 08, France;* [2]*Centro de Control de Cancer, Calle 3A la Concordia, San Cristobal, Venezuela.*

**Summary** A screening programme for early gastric cancer was introduced in the state of Tachira, Venezuela, in 1980. Screening was performed by photofluorography, using two mobile units. The efficacy of this programme in reducing mortality from stomach cancer was evaluated by means of a case–control study. Cases were 241 individuals who died from stomach cancer in the period 1985–89. Ten live controls per case were drawn from the electoral rolls, matched by sex, age and residence. Exposure to the screening examination of cases and controls was assessed through individual linkage with the programme's centralised database. After the exclusion of examinations occurring within the 6 months preceding the case's diagnosis, the odds ratio (OR) of dying from stomach cancer for those screened was 1.26 (CI 0.83–1.91) and the OR in females was lower than in males: 0.77 (CI 0.33–1.78) and 1.52 (CI 0.94–2.47) respectively. Odds ratios associated with years since last test and number of tests did not differ significantly from 1. These results show the inefficacy of the programme in reducing mortality from gastric cancer in the area. In an attempt to determine whether this result was due to selection bias, an analysis restricted to subjects who had been screened at least once was performed. When examinations occurring after an index date at various intervals before the case's diagnosis were excluded, the screening test appeared to protect from death, although confidence intervals of the odds ratios are large, for example OR = 0.47 (CI 0.24–0.98) when excluding tests within 1 month.

Stomach cancer, despite a decline in incidence almost everywhere, remains the second most common cancer world-wide, after lung cancer (Parkin *et al.*, 1993). In Japan, where stomach cancer is a major public health problem, there has been an extensive attempt to reduce mortality by early detection and treatment. The population screening programme aims to examine 30% of the population aged 40 or over each year by photofluorography (Oshima, 1988). The test aims at detecting the disease at an early stage, when cancer is confined to the gastric mucosa and submucosa. The Japanese programmes were introduced as a community service, without any formal randomised trial, so that evaluation of their success has depended upon analyses of time trends in incidence and mortality, comparisons of gastric cancer mortality in screened versus unscreened populations and case-control analyses (see reviews by Oshima, 1988; Hisamichi, 1989; Hisamichi *et al.*, 1991). The problem of interpreting the results of non-randomised evaluations has meant that there is still some scepticism concerning the applicability of mass screening for gastric cancer elsewhere (Chamberlain *et al.*, 1986; Miller *et al.*, 1990).

In common with other mountainous parts of South and Central America, the state of Tachira in the western part of Venezuela has high rates of gastric cancer – age-standardised mortality rates in 1982–84 were 49.6 per $10^5$ in men and 34.1 in women, compared with 40.8 and 19.0 in men and women, respectively, in Japan in 1987–88 (Aoki *et al.*, 1992). A screening programme was started in 1980, by means of the established Japanese methodology of six film indirect photofluorography using double contrast. The objective was to examine as large a proportion of the population aged 35 or over as possible at intervals of 1–2 years with the aim of reducing mortality from gastric cancer. Two mobile screening units were used, moving to different localities; they were installed in a locality and subjects were invited for screening by rural nurses, using population lists for health centres close to the screening unit's location.

In the years 1981–89 some 114,000 examinations were performed, resulting in the detection of 445 cancers. In this paper, we evaluate the success of the programme in preventing death from gastric cancer, using a case–control analysis.

Correspondence: P. Pisani.
Received 5 July 1993; and in revised form 21 December 1993.

### Materials and methods

A total of 250 individuals who were resident in Tachira State and who died from gastric cancer during the period 1985–89 formed the case group. These represented almost all of the deaths from gastric cancer in this period for which the death certificate diagnosis could be confirmed by tracing the relevant clinical records in hospitals or medical centres. The date of diagnosis, as noted in the clinical record, was abstracted for each case, together with information on the basis of diagnosis and the presence or absence of clinical metastases. Seventy-four per cent of the cases had been histologically confirmed, 8.3% had a clinical diagnosis only, and the remainder were diagnosed by radiology, cytology or other means.

Ten live controls were matched to each case; they were drawn from the electoral registers of the case's polling district for the year of death of the case, matching on sex and age (± 3 years). Polling districts are defined as the place where one votes, and as they cover small areas the sampling procedure entailed matching by neighbourhood. Electoral rolls include all residents aged 18 +. Nine cases were excluded because of an error in selecting the controls (controls of the wrong sex were included), leaving 241 match sets (241 cases and 2410 controls) for the analysis.

For each case and control subject, the centralised database of the screening programme was searched; if found, their complete screening history (date and results of radiological, endoscopic and biopsy examinations) was abstracted.

### *Exposure definition*

In the screening database 105 cases and 376 controls could be traced, corresponding to a crude prevalence of 43% of case subjects and 16% of controls ever having been screened.

For 40 of the cases there was a malignant diagnosis at a screening examination which took place before the date of diagnosis in the clinical record, and the date of diagnosis was advanced to the date of the first biopsy which resulted in malignancy.

Four index dates were used in the definition of exposure to the screening programme:

A. the date of diagnosis of the case (including the test, if any, which resulted in the diagnosis of gastric cancer);
B. 1 month before the date of diagnosis of the case;
C. 6 months before the date of diagnosis of the case;

D. 1 year before the date of diagnosis of the case.
Any examination which was performed after the index date was excluded, irrespective of the case control status.

Odds ratios (ORs) of dying from gastric cancer, given exposure to the screening programme, were estimated through conditional logistic regression for matched data (Breslow & Day, 1980); consequently, ORs were adjusted by sex, age and neighbourhood. ORs greater than 1 indicate a higher risk of dying from gastric cancer if screened, and ORs lower than 1 indicate a lower risk.

### Results

Table I shows the distribution of cases and controls by screening history (ever/never). The crude prevalence of controls ever examined is 11.0% with no exclusion of tests up to diagnosis of the case. It is obvious that, among the cases, an excess number of examinations took place close to their date of diagnosis; passing from definition A to B the prevalence of exposed cases decreases dramatically from 35.3% to 19.9%, while the corresponding figure for controls, 11.0%, does not change.

The ORs associated with the four exposure definitions to the screening programme are also shown in Table I. A significant OR of 4.82 (CI 3.54–6.57) is observed for screened subjects versus those not screened when all tests are considered (exposure defined as in A). Most of this excess risk is confined to examinations close to the diagnosis: the OR decreases to 2.08 (CI 1.96–2.96) when excluding tests within 1 month before diagnosis (exposure B), and to 1.26 (CI 0.83-1.91) when excluding tests within 6 months (exposure C); the OR does not change further when excluding examinations within 1 year before diagnosis: OR = 1.32 (CI 0.86–2.03) (exposure D).

The risk associated with screening is analysed in greater detail in Table II for exposure as defined in C. Most of the excess risk is confined to men: their OR is 1.52 (CI 0.94–2.47), while the risk among women is 0.77 (CI 0.33–1.78); ORs are higher for men than for women whatever the exposure definition adopted (data not shown).

No excess risk was detected for examination within 3 years before diagnosis, while a significant OR of 1.81 (CI 1.02–3.21) was observed for examinations occurring 3 years or more before; this excess risk is confined to males: the OR is 2.18 (CI 1.11–4.27).

The results presented so far clearly show that those who attended the screening programme are at a higher risk of dying from gastric cancer than the general population; quite possibly many attend for screening because of the presence of symptoms. In order to avoid this selection bias, an analysis limited to those subjects who had had at least one screening test was performed. Any screening test, irrespective of when it was performed, would define the subject as eligible for this analysis. The only tests excluded in defining the study population were those occurring among cases after the date of diagnosis. Exposure was then defined as above, thus excluding examinations performed after the index date. Table

**Table I** Distribution of cases and controls by exposure to screening and odds ratio (OR) estimates, screened *vs* not screened, 95% confidence limits. Total number of cases and controls is 241 and 2410 respectively

| *Index date* | *Screened No.* | *Screened %* | *Not screened* | *OR* | *CI* |
|---|---|---|---|---|---|
| A At diagnosis | | | | | |
| Cases | 85 | 35.3 | 156 | 4.82 | (3.54–6.57) |
| Controls | 265 | 11.0 | 2145 | | |
| B One month before diagnosis | | | | | |
| Cases | 48 | 19.9 | 193 | 2.08 | (1.96–2.96) |
| Controls | 265 | 11.0 | 2145 | | |
| C Six months before diagnosis | | | | | |
| Cases | 30 | 12.4 | 211 | 1.26 | (0.83–1.91) |
| Controls | 248 | 10.3 | 2162 | | |
| D Twelve months before diagnosis | | | | | |
| Cases | 28 | 11.6 | 213 | 1.32 | (0.86–2.03) |
| Controls | 222 | 9.2 | 2188 | | |

**Table II** Odds ratios (ORs) and confidence intervals (CIs) associated with exposure to screening tests, time since last test and number of tests. Exposure defined as in C – tests within 6 months excluded. Total number of cases and controls entering the analysis is 241 and 2410 respectively

| *Exposure C* | *No. of cases* | *OR (CI) All subjects* | *OR (CI) Males* | *OR (CI) Females* |
|---|---|---|---|---|
| Not screened[a] | 211 | 1 | 1 | 1 |
| Screened | 30 | 1.26 (0.83–1.91) | 1.52 (0.94–2.47) | 0.77 (0.33–1.78) |
| Years since last test | | | | |
| Never[a] | 211 | 1 | 1 | 1 |
| <3 | 14 | 0.94 (0.53–1.67) | 1.16 (0.60–2.23) | 0.54 (0.16–1.81) |
| 3+ | 16 | 1.81 (1.02–3.21) | 2.18 (1.11–4.27) | 1.15 (0.37–3.53) |
| Number of tests | | | | |
| 0[a] | 211 | 1 | 1 | 1 |
| 1 | 27 | 1.27 (0.82–1.97) | 1.55 (0.94–2.55) | 0.76 (0.31–1.88) |
| 2+ | 3 | 1.12 (0.33–3.75) | 1.33 (0.30–5.93) | 0.79 (0.10–6.37) |

[a]Reference category.

III presents the results of this analysis, in which the index date is 1 month before the diagnosis of the case (definition B). The overall risk for subjects screened before the index date is 0.47 (CI 0.24–0.98) relative to those not screened, and is statistically significant. The protective effect of the test appears to be constant by time since last examination, but confidence intervals are large because of the relatively small numbers involved – the number of discordant sets contributing to this analysis is 64.

The same analysis was repeated with the third exposure definition (C), the results of which are presented in Table IV. The exclusion of recent examinations does not yield higher estimates of the screening efficacy: the OR for those classified as screened versus those not screened is 0.25 (CI 0.12–0.51); the beneficial effect is statistically significant whether the last test occurred within the last 3 years or earlier.

### Discussion

This study was designed to get a first insight into the impact of the screening programme for gastric cancer in Tachira State, Venezuela, which has been going on for over 10 years. The programme is expensive and the evaluation of its effectiveness was of high priority. Moreover, other countries in the same areas are currently considering the introduction of similar progammes; consequently, it is important to know the results of the only experience conducted under similar conditions. Our study shows that the programme in Tachira State has failed to reduce mortality from gastric cancer because of the low coverage of the population.

The main defect of case–control studies in the evaluation of the efficacy of screening is the effect of selection bias. Cases and controls are drawn from a population with access to screening, and the evaluation estimates the risk of disease in those who attend for screening versus those who do not. The fact that risk of the disease outcome under study (here, death from cancer) differs in the two groups irrespective of screening is a potent source of bias (Connor *et al.*, 1991; Moss, 1991; Weiss *et al.*, 1992). Selection bias is a problem in the present study, since there is a high risk of death in those accepting screening, presumably because they are using the screening programme as a diagnostic service for symptoms of gastric cancer. Such a finding might have been anticipated from the low prevalence of screening (some 12.4% of those in the age groups at risk of death from gastric cancer). In the years 1981–89, only some 16% of the cancers detected by screening were defined as 'early'.

**Table III** Cases and controls who had at least one screening test. Exposure defined as in B – tests within 1 month excluded

| | *All subjects* | | | |
|---|---|---|---|---|
| | *Cases* | *Controls* | *OR* | *CI* |
| Not screened[a] | 37 | 111 | 1 | |
| Screened | 48 | 264 | 0.47 | (0.24–0.98) |
| Total | 85 | 375 | | |
| Years since last test | | | | |
| Never[a] | 37 | 111 | 1 | |
| <3 | 34 | 173 | 0.48 | (0.24–0.97) |
| 3+ | 14 | 91 | 0.45 | (0.19–1.10) |
| Total | 85 | 375 | | |

[a]Tests occurred after the index date; reference category.

**Table IV** Cases and controls who had at least one screening test, irrespective of the index date. Exposure defined as in C – tests within 6 months excluded

| | *All subjects* | | | |
|---|---|---|---|---|
| | *Cases* | *Controls* | *OR* | *CI* |
| Not screened[a] | 55 | 129 | 1 | |
| Screened | 30 | 246 | 0.25 | (0.12–0.51) |
| Total | 85 | 375 | | |
| Years since last test | | | | |
| Never[a] | 55 | 129 | 1 | |
| <3 | 14 | 151 | 0.15 | (0.06–0.39) |
| 3+ | 16 | 95 | 0.40 | (0.17–0.95) |
| Total | 85 | 375 | | |

[a]Tests occurred after the index date; reference category.

Experience in western countries suggests that persons of higher social class are more likely to make use of preventive services such as screening; if this were so for the gastric cancer screening programme in Venezuela, we might expect this selection bias to produce an apparent protective effect of screening, since gastric cancer is less common in the higher social classes. On the other hand in many developing countries, a large part of the population has limited access to health facilities, especially if resident in rural areas (because health centres are concentrated in cities and public transport is almost non-existent and because health care is not free); thus the screening units when installed in villages are the only chance of having a diagnostic examination for those affected by gastric symptoms of a variety of disorders, including cancer. In western countries self-selection for screening does not operate in a predictable direction. In the Health Insurance Plan of the Greater New York Breast Cancer Screening Trial (HIP Trial), among women allocated to the screening arm those who accepted the examination were at higher risk of breast cancer than those who refused it (Friedman & Dubin, 1991). In a case–control study of mortality from breast cancer, Palli *et al.* (1986) found that women referring themselves to an early diagnosis clinic were at twice the risk of dying from breast cancer in comparison with those who did not. On the contrary, in the UK trial of early detection of breast cancer, women who accepted screening were at lower risk of dying from breast cancer than those who did not (Moss, 1991).

The over-representation of high-risk people among the screened in our study could theoretically be corrected by matching cases and controls for the presence of symptoms of the disease at the time of diagnosis. Information on the presence of symptoms in screen-detected cases at the time of diagnosis was not available through the programme and obviously could not be traced retrospectively. However, it should be emphasised that control of selection bias by adjusting for symptoms is likely to be only a theoretical option, as symptoms of gastric cancer are non-specific and common to other gastric disorders such as chronic atrophic gastritis and intestinal metaplasia, the prevalence of which is very high (45%) in populations of South America showing high incidence rates of gastric cancer (Correa *et al.*, 1990).

Consequently, selection bias was controlled for through matching by age and residence (which allows similar probability of having access to the examination for cases and controls), through the exposure definition, and finally by limiting the analysis to those who had been screened at least once.

Interestingly, in an investigation which simulated the results of case–control studies to evaluate the efficacy of screening for breast cancer, and which took advantage of the results of the HIP Trial, it was shown that control by a variety of confounders, including an indicator of symptoms, did eliminate the effect of selection bias in the 'population-based' study design; on the other hand it did not materially affect the estimates of the ORs when the study was limited to screened women (Friedman & Dubin, 1991).

The present analysis does provide some suggestion of benefit from screening in the observation that, when tests within 6 months of diagnosis are excluded, the risk of death in the 3 years following a negative test is about half that when the last test was more than 3 years earlier.

In a similar case–control study in the town of Nose, Japan, Oshima *et al.* (1986) found an OR for screened versus unscreened subjects of 0.52 in men and 0.49 in women when tests within 1 year of diagnosis were excluded. Tests within 2

years of diagnosis (excluding the diagnostic test) were associated with a very low OR (0.17) compared with earlier tests (0.65). The difference from the results in Tachira are presumably related to the greater intensity of screening – some 67% of the controls from the Nose population had had one or more tests, and 23/56 (41%) of cases detected by screening were early gastric cancer. Nevertheless, there was some evidence of attendance related to symptoms – ORs (in men only) were higher when tests within a year of diagnosis were included.

One other case-control study has been performed in Japan (Fukao *et al.*, 1987), comparing screening histories before diagnosis in 367 cases of advanced stomach cancer with controls matched for sex, age and residence (precinct). A protective effect was observed up to 3 years since the last negative test; this was significant (OR = 0.34, 95% CI 0.25–0.48) in the first year after screening. The prevalence of screening was high in this population – 77% of controls had been screened at some time, 60% in the previous year.

The questions that remain concerning the efficacy of gastric cancer screening cannot be resolved by further observational studies, which do not address the effects of selection bias (positive or negative) on the outcome. Adoption of gastric cancer screening as public health policy in Japan means that the opportunity for a randomised trial, with a non-screened control group, is no longer feasible. One randomised trial is in progress (Hisamichi *et al.*, 1991), in which 39 municipalities were randomised in two groups. People resident in municipalities belonging to the first group and aged 50, and people from the second group of residence aged 60, form the intervention group. They were sent a personal invitation to attend for screening while the rest of the population was offered the examination in the usual way. Although there was some difference in screening attendance between the intervention group and the rest of the population in the year following the intervention, this had declined to a very small differential by the second year, and it is inconceivable that a demonstrable difference in mortality will result. Outside Japan, we are aware only of mass screening by radiography in Tachira State, Venezuela, and on a smaller scale in Ecuador. These programmes are, at present, small and with insufficiently high population coverage, so that even a randomised controlled trial would have insufficient power to demonstrate the likely degree of efficacy. Only a substantial improvement of the population coverage would permit a proper evaluation.

We wish to thank Mrs D. Vargas, Mrs C. Ramirez, Mr E. Meza, Mr J. Rodriguez and all the social workers of the Cancer Control Centre in San Cristobal for their valuable collaboration, Dr E. Buiatti and Dr N. Muñoz for useful discussions, and Miss M. Geesink for typing the manuscript.

### References

AOKI, K., KURIHARA, M., HAYAKAWA, N. & SUZUKI, S. (1992). *Death Rates for Malignant Neoplasms for Selected Sites by Sex and Five-Year Age Group in 33 Countries, 1953–57 to 1983–87*. UICC: Geneva.

BRESLOW, N.E. & DAY, N.E. (1980). *Statistical Methods in Cancer Research, Vol. 1, The Analysis of Case-Control Studies* (IARC Scientific Publications No. 32). International Agency for Research on Cancer: Lyon.

CHAMBERLAIN, J., DAY, N.E., HAKAMA, M., MILLER, A.B. & PROROK, P.C. (1986). UICC workshop of the project on evaluation of screening programmes for gastrointestinal cancer. *Int. J. Cancer*, **37**, 329–334.

CONNOR, R.J., PROROK, P.C. & WEED, D.L. (1991). The case-control design and the assessment of the efficacy of cancer screening. *J. Clin. Epidemiol.*, **44**, 1215–1221.

CORREA, P., HAENSZEL, W., CUELLO, C., ZAVALA, D., FONTHAM, E., ZARAMA, G., TANNENBAUM, S., COLLAZOS, T. & RUIZ, B. (1990). Gastric precancerous process in a high risk population: cross-sectional studies. *Cancer Res.*, **50**, 4731–4736.

FRIEDMAN, D.R. & DUBIN, N. (1991). Case-control evaluation of breast cancer screening efficacy. *Am. J. Epidemiol.*, **133**, 974–984.

FUKAO, A., HISAMICHI, S. & SUGAWARA, N. (1987). A case-control study on evaluating the effect of mass screening on decreasing advanced stomach cancer. *J. Jpn Soc. Gastroenterol. Mass Survey*, **75**, 112–116.

HISAMICHI, S. (1989). Screening for gastric cancer. *World J. Surg.*, **13**, 31–37.

HISAMICHI, S., FUKAO, A., SUGAWARA, N. & 5 others (1991). Evaluation of mass screening programme for stomach cancer in Japan. In *Cancer Screening*, Miller, A.B., Chamberlain, J., Day, N.E., Hakama, M. & Prorok, P.C. (eds), pp. 357–370. Cambridge University Press (on behalf of UICC): Cambridge.

MILLER, A.B., CHAMBERLAIN, J., DAY, N.E., HAKAMA, M. & PROROK, P.C. (1990). Report on a workshop of the UICC project on evaluation of screening for cancer. *Int. J. Cancer*, **46**, 761–769.

MOSS, S.M. (1991). Case control studies of screening. *Int. J. Epidemiol.*, **20**, 1–6.

OSHIMA, A. (1988). Screening for stomach cancer: the Japanese program. In *Screening for Gastrointestinal Cancer*, Chamberlain, J. & Miller, A.B. (eds), pp. 65–70. Hans Huber Publishers (for UICC): Toronto.

OSHIMA, A., HIRATA, N., UBUKATA, T., UMEDA, K. & FUJIMOTO, I. (1986). Evaluation of a mass screening program for stomach cancer with a case-control study design. *Int. J. Cancer*, **38**, 829–833.

PALLI, D., ROSSELLI DEL TURCO, M., BUIATTI, E., CARLI, S., CIATTO, S., TOSCANI, L. & MALTONI, G. (1986). A case control study of the efficacy of a non-randomized breast cancer screening program in Florence (Italy). *Int. J. Cancer*, **38**, 501–504.

PARKIN, D.M., PISANI, P. & FERLAY, J. (1993). Estimates of the worldwide incidence of eighteen major cancers in 1985. *Int. J. Cancer*, **41**, 184–197.

WEISS, N.S., MCKNIGHT, B. & STEVENS, N.G. (1992). Approaches to the analysis of case-control studies of the efficacy of screening for cancer. *Am. J. Epidemiol.*, **135**, 817–823.